# Flow cytometric analysis of innate lymphoid cells: challenges and solutions

**DOI:** 10.3389/fimmu.2023.1198310

**Published:** 2023-09-22

**Authors:** Mona Sadeghalvad, Davit Khijakadze, Mona Orangi, Fumio Takei

**Affiliations:** ^1^ Terry Fox Laboratory, British Columbia Cancer, Vancouver, BC, Canada; ^2^ Interdisciplinary Oncology Program, University of British Columbia (UBC), Vancouver, BC, Canada; ^3^ Department of Pathology and Laboratory Medicine, University of British Columbia (UBC), Vancouver, BC, Canada

**Keywords:** innate lymphoid cells, ILC1, ILC2, ILC3, flow cytometry, analysis

## Abstract

**Introduction:**

The three groups of helper innate lymphoid cells (ILCs), namely ILC1, ILC2 and ILC3, have been identified by flow cytometry by combinations of cell surface markers. Here, we review various ways ILCs are currently identified, focusing on potential problems and their solutions. The first step to identify all ILCs is to exclude other lymphocytes and myeloid cells by their lineage-specific markers (Lin). However, the Lin cocktail varies in various studies, and the definition of Lin- population containing ILCs is often ambiguous, resulting in contamination of Lin^+^ cells, particularly T cells.

**Method:**

We have designed combinations of cell surface markers to identify ILC populations in various tissues of B6 mice by flow cytometry. To minimize T cell contamination, TCR/CD3ϵ antibodies were used separately from the Lin cocktail. ILCs identified by surface markers are confirmed by the expression of the transcription factors GATA3, RORγt, T-bet and Eomes.

**Result:**

ILC1s in the B6 mouse liver are identified by Lin-NKp46^+^NK1.1^+^TCR/CD3ϵ^−^CD49a^+^CD49b^−^. However, defining ILC1s in other tissues remains a challenge. ILC2s in the lung are identified by Lin^−^TCR/CD3ϵ^−^ Thy1^+^CD127^+^ST2^+^ whereas ILC2s in the small intestine and liver are identified by Lin^−^TCR/CD3ϵ^−^Thy1^+^GATA3^+^RORγt^−^. ILC3s in B6 mouse spleen, liver, lung and small intestine are identified by Lin^−^TCR/CD3ϵ^−^ Thy1^+^CD127^+^RORγt^+^.

**Discussion:**

The ILC population is heterogeneous and the strategies to identify ILCs have to be designed for each ILC population and tissue. Excluding T cells in all cases is crucial, and a combination of transcription factors GATA3, RORγt, T-bet, and Eomes should be used to identify ILCs. Using CD3ϵ/TCRs in a different fluorochrome not in Lin cocktail minimizes contamination of T cells specifically identify individual ILC populations in various tissues.

## 1 Introduction

The family of cytokine-producing helper innate lymphoid cells (ILCs) consists of three groups, namely, ILC1, ILC2, and ILC3 (1). ILCs do not express unique markers, and they can only be distinguished from each other and other lymphocytes by combinations of markers. All ILCs are negative for mature hematopoietic cell lineage surface markers (Lin^−^). For mouse ILCs, the Lin cocktail typically includes Ter119 for erythroid cells, Gr-1 for granulocytes, CD11c for dendritic cells, CD11b for monocytes/macrophages, CD19 for B cells, and CD3ϵ/TCR for T cells (2). With this Lin cocktail, Lin^−^ cells include all ILCs and also a subset of NK cells that is CD11b negative. To avoid NK cell contamination, NK1.1 is used in the lineage cocktail or in separate fluorochrome to exclude NK cells (3). However, the separation between Lin^+^ and Lin^−^ cells is often not clear due to the presence of cells expressing an intermediate level of Lin markers. Because the Lin^−^ gate is often arbitrarily set, some Lin^+^ cells, particularly T cells, can be included in the Lin^−^ population. ILCs within the Lin^−^ population can be further identified by CD127 and/or Thy1 expression. However, some ILC1s do not express Thy1 and CD127, while some NK cells express Thy1 and CD127. Currently, there is no simple gating strategy to identify all ILCs by combinations of cell surface markers, and the Lin cocktail and subsequent gating for ILCs have to be designed for each ILC population.

Each ILC population is heterogeneous, and cell surface marker expression varies among ILCs in different tissues (4–6), making it difficult to identify ILCs in various tissues by cell surface markers alone. ILCs can also be identified by the expression of the transcription factors T-bet, Eomes, GATA3, and RORγt. ILC2s are T-bet^−^Eomes^−^GATA3^hi^RORγt^−^, ILC3s are Eomes^−^GATA3^lo^RORγt^+^, and ILC1s and NK cells are T-bet^+^RORγt^−^ (7, 8). The distinction between NK cells and ILC1s is complicated by the heterogeneity of ILC1s. ILC1s in the liver are Eomes^−^GATA3^−^, while salivary gland ILC1s express Eomes and T-bet. Intestinal and thymic ILC1s are Eomes^−^T-bet^+^GATA3^+^ (9). As T cells also express these transcription factors, it is critical to exclude T cells from this analysis. Recent reports have also shown that ILC progenitors expressing those transcription factors are found in various tissues (10), further complicating ILC analyses.

While the transcription factor staining can confirm the identity of individual ILC populations, it requires fixing and permeabilization of cells and cannot be applied to isolate live ILCs. There are fluorescent reporter mice that can be used to identify ILCs without fixing/permeabilization of cells, including reporters for GATA3 (11), IL-5 and IL-13 (12), RORγt (13), and NKp46 (14). Because those genes are not specific to ILCs but are also expressed in some other lymphocytes, combinations of other cell surface markers are required to identify individual ILC populations.

Here, we discuss problems with the ways ILCs are currently identified by flow cytometry and how individual ILC populations in the lung, liver, and small intestine (SI) can be identified by combinations of cell surface markers and transcription factors without contamination of other lymphocytes.

## 2 Materials and methods

### 2.1 Mice

C57BL/6J (B6) mice were bred in-house and purchased from breeders of Jackson Laboratory (Bar Harbor, ME, USA). *Rorc(*γ*t)*-eGFP reporter mice (JAX stock #007572) and *Rag1* ^−/−^ mice (JAX stock #002216) on a B6 background were originally purchased from Jackson Laboratory. Homozygous *Rorc(*γ*t)*-eGFP mice were crossed with B6 mice to generate heterozygous *Rorc(*γ*t)*-eGFP mice. All mice were maintained in the British Columbia Cancer Research Centre animal facility under specific pathogen-free conditions. The use of these mice was approved by the animal committee of the University of British Columbia and in accordance with the guidelines of the Canadian Council on Animal Care.

### 2.2 Tissue processing and leukocyte preparation

All tissues including the liver, lung, spleen, and SI were processed as described previously in detail (15). Mice were anesthetized with isoflurane (5%) and then euthanized with CO_2_ until respiratory arrest. Tissues were collected in a tube containing 5 ml of Dulbecco’s modified Eagle medium (DMEM) with 2% fetal bovine serum (FBS).

#### 2.2.1 Liver

Livers were mashed and passed through 40-µm cell strainers (BD Falcon or Sarstedt) with 10 ml of DMEM (Thermo Fisher Scientific, Waltham, MA, USA; #11995073) + 10% (v/v) FBS, and the strainers were washed with 15 ml of the same media. After centrifugation (4°C, 3 min, 30 × *g*) for hepatocyte sedimentation, the supernatant was collected and centrifuged (4°C, 5 min, 300 × *g*). The pellet was resuspended in 16 ml of 40% Percoll. After centrifugation (room temperature (RT), 20 min, 1,400 × *g*), the pellet resuspended in 3 ml of red blood cell (RBC) lysis buffer (ammonium chloride, NH_4_Cl; MilliporeSigma, St. Louis, MO, USA; cat. no. A9434-500G; sodium bicarbonate, NaHCO_3_; Thermo Fisher Scientific, cat. no. BP328-500; EDTA disodium salt, Thermo Fisher Scientific, cat. no. BP120-1, pH 7.2–7.4).

#### 2.2.2 Spleen

Spleens were mashed through 70-µm strainers in 5 ml phosphate-buffered saline (PBS) + 2% FBS. Strainers were washed with 5 ml of the same buffer. After centrifugation (4°C, 5 min, 400 × *g*), the supernatant was discarded, and RBCs were lysed using RBC lysis buffer.

#### 2.2.3 Lung

Lungs were minced in a 10-cm petri dish by using a razor and then placed in a tube containing 5 ml of digestion buffer (DMEM + 10% FBS + 142.5 U/ml Collagenase IV, Thermo Fisher Scientific, cat. no. 17104019; 118.05 KU/ml of DNase I from bovine pancreas, MilliporeSigma, cat. no. 11284932001). Tubes were incubated for 25 min at 37°C in a shaker at 200 rpm. Digested tissues were mashed and passed through 70-µm cell strainers by adding 5 ml DMEM + 10% FBS. After centrifugation (4°C, 5 min, 400 × *g*), the supernatant was discarded, and the pellet was resuspended in 5 ml of 36% Percoll and centrifuged at RT, 20 min, 650 × *g*. RBCs were lysed using RBC lysis buffer.

#### 2.2.4 Small intestine

The SIs were extensively washed with intestine wash buffer (Ca^2+^/Mg^2+^-free (CMF) HBSS + 15 mM HEPES/pH = 7.2) and were cut into ~1-cm pieces after discarding the fat and Peyer’s patches. With the use of surgical scissors, each fragment was cut open longitudinally. Then, the luminal contents were rinsed three times using an intestine wash buffer. The intestinal epithelial cells were removed by incubation in CMF/EDTA/FBS buffer (HBSS + 15 mM HEPES + 10% FBS + 2.5 mM EDTA + 1 mM DTT/pH = 7.2) for 20 min at 37°C in a shaker at 250 rpm. The tubes were shaken manually 10 times, and then the contents were passed through sieves and rinsed with wash buffer. This step could be repeated for two more times. Tissues were digested using 100 U/ml of Collagenase VIII (Sigma-Aldrich, cat. no. C2139-500MG) and 9.41 U/ml of DNase I from bovine pancreas (Millipore Sigma, cat. no. 11284932001). Digestion buffer was prepared in DMEM containing 10% FBS. Tissue digestion was performed in a shaker for 25 min at 37°C and 5% CO_2_. The contents were filtered through a 100-μm cell strainer, and 10 ml of intestine washing buffer was added. After centrifugation (4°C, 5 min, 400 × *g*), the pellet was resuspended in 5 ml of PBS containing 2% FBS and filtered through 70-μm and then 40-μm cell strainers. Cells were centrifuged (4°C, 5 min, 400 × *g*), and then RBCs were lysed using RBC lysis buffer.

After adding RBC lysis buffer, cells were incubated for 5 min at RT. DMEM at a volume of 5 ml with 2% FBS was added and centrifuged (4°C, 5 min, 400 × *g*). The supernatant was discarded, and the pellet was resuspended in 1 ml PBS + 2% FBS and then transferred to a 5-ml flow tube through a 35-μm strainer cap. Cells were then stained with antibodies in PBS containing 2% FBS.

### 2.3 Intracellular cytokine staining

Leukocytes were incubated at 37°C for 3 hours in 500 µl of Roswell Park Memorial Institute (RPMI) 1640 media containing 10% FBS, 100 U/ml of P/S, 50 mM of 2-ME, Brefeldin A (Golgi Plug, BD Biosciences, San Jose, CA, USA), 30 ng/ml of phorbol 12-myristate 13-acetate (PMA; Sigma, P1585), and 500 ng/ml of ionomycin (Sigma, 10634). Intracellular cytokine staining was performed after the incubation and surface staining using Cytofix/Cytoperm Fixation/Permeabilization Solution kit (BD Biosciences) according to the manufacturer’s protocol. Intracellular GATA3, RORγt, T-bet, and Eomes staining were carried out similarly without preincubation using Foxp3/Transcription Factor Staining Buffer Set (Thermo Fisher Scientific) according to the manufacturer’s protocol. Simultaneous intracellular RORγt and cytokine staining were performed using Foxp3/Transcription Factor Staining Buffer Set according to the manufacturer’s protocol.

### 2.4 Antibodies and flow cytometry

Single-cell suspensions were incubated with anti-mouse CD16/32 antibody (clone 2.4G2) to block non-specific binding to Fc receptors before surface staining. Antibodies used in this study are listed in **Table 1**. Flow cytometry analysis was performed on a BD Fortessa flow cytometer and FACSDiva software (BD Biosciences). Flow cytometry data were analyzed using FlowJo 8.7. GraphPad Prism 7 was used for data analysis. Data shown in the figures are mean ± SEM.

**Table 1:** Antibody list for flow cytometry.

Antigen	Clone	Fluorochrome	Brand	Dilution
Viability dye	—	eFIuor780	Thermo Fisher	500
CD45	30-F11	BV510	BD Biosciences	100
CD127	A7R34	PE	Thermo Fisher	250
CD127	A7R34	AF700	Thermo Fisher	80
NKP46	29A1.4	eFluor450	Thermo Fisher	250
NKP46	29A1.4	PerCPeF710	Thermo Fisher	200
NKP46	29A1.4	AF700	Invitrogen	
CD19	ID3	eFluor450	Thermo Fisher	750
LY6G and LY6C (GR-1)	R86-8C5	eFluor450	Thermo Fisher	1000
TCRβ	H57-597	PE	BioLegend	250
TCRβ	H57-597	FITC	BD Biosciences	250
TCRγδ	eBioGL3	PE	BD Biosciences	750
TCRγδ	GL3	FITC	BD Biosciences	750
CD3	145-2C11	PE	BD Biosciences	125
CD3	145-2C11	FITC	Thermo Fisher	250
GATA3	TWAJ	APC	Thermo Fisher	50
Rorγt	B2D	PE	Thermo Fisher	50
Eomes	Dna11mag	PerCPeF710	Thermo Fisher	50
T-bet	eBio4B10	PECY7	Thermo Fisher	50
Ter119	TER119	eFluor450	Thermo Fisher	250
Thy1.2	33-2.1	BV605	BD Biosciences	250
B220	RA3-6B2	eFluor450	Thermo Fisher	750
NK1.1	PK136	eFluor450	Thermo Fisher	250
NK1.1	PK136	APC	BD Biosciences	150
CD11c	N418	eFluor450	Thermo Fisher	750
CD11b	M1/70	eFluor450	Thermo Fisher	750
ST2	RMST2-2	PECY7	Thermo Fisher	80
CD49a	Ha31/8	BV711	BD Biosciences	250
CD49b	DX5	AF647	BioLegend	250
CCR6	140706	BV711	BD Biosciences	250
IFNγ	XMG1.2	PECY7	Thermo Fisher	50
IL-5	TRFK5	APC	BD Biosciences	100
IL-13	eBio13A	PECY7	Thermo Fisher	50
Rorγt	B2D	PE	Thermo Fisher	50
IL-17	TC11-18H10	PE	BD Biosciences	50
IL-22	1H8PWSR	PerCPeF710	Thermo Fisher	50

## 3 Results

### 3.1 Analysis of ILC1

**Figure 1:**
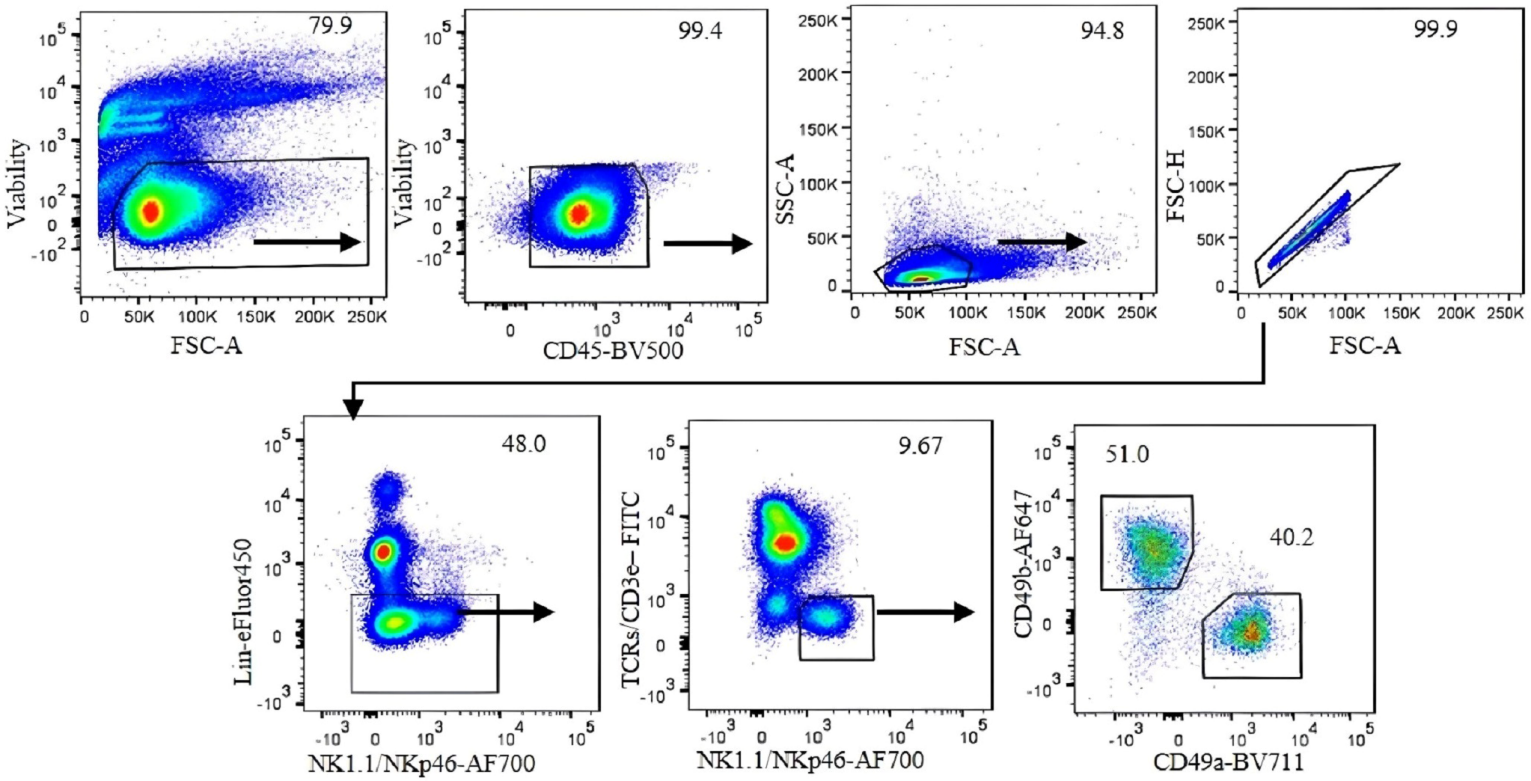
Gating strategy for analysis of liver ILC1 in C57BL/6 mice. Gating was applied on live CD45^+^ leukocytes. Lymphocytes were gated with the forward scatter/side scatter (FSC/SSC) method, followed by gating on single cells using FSC-A/FSC-H. Negative lineage cells were gated to exclude erythrocytes, myeloid cells, and B cells. NK1.1- and NKp46-positive, T-cell marker-negative cells were gated. CD49b and CD49a markers were used to define NK cells and ILC1, respectively. Numbers indicate percentages of cells in each gate.

To accurately identify ILC1s in the liver under steady-state conditions, we performed 12-color flow cytometry (**Table 2**) and sequential gating analysis (**Figure 1**). Our lineage cocktail contained Ter119, Gr-1, CD220, and CD19 to exclude erythrocytes, granulocytes, and B cells. We used a T-cell antibody cocktail including TCRβ, TCRγδ, and CD3ϵ in a separate color to avoid NKT cell contamination. We used NK1.1 and NKp46 (NCR1) as shared markers for identifying NK cells and ILC1s. It is important to note that NK1.1 is expressed in B6 and SJL strains but not in other strains (16).

**Table 2:** Antibody panel for ILC1, ILC2, and ILC3 identification.

ILC1 panel											
FITC	PerCPeF710	AF647	AF-700	R780	eFlour450	BV510	BV605	BV650	BV711	PE	PECy7
CD3ϵ, TCRβ, TCRγδ	Eomes	CD49b	NK1.1, NKp46	Viab	Ter119, Gr-1 CD19, B220	CD45	Thy1.2	CD160	CD49a	CD127	T-bet
ILC2 panel											
FITC	PerCPeF710	R670	AF-700	R780	eFlour450	BV510	BV605	BV650	BV711	PE	PECy7
CD3ϵ, TCRβ, TCRγδ	ST2	GATA3	–	Viab	Ter119, Gr-1 CD19, B220, NK1.1, NKp46	CD45	Thy1.2	–	–	Rorγt	CD127
ILC3 panel											
FITC	PerCPeF710	R670	AF-700	R780	eFlour450	BV510	BV605	BV650	BV711	PE	PECy7
CD3ϵ, TCRβ, TCRγδ	ST2	GATA3	CD127	Viab	Ter119, Gr-1 CD19, B220	CD45	Thy1.2	–	–	Rorγt	NK1.1 (or in Lin cocktail)

**Figure 2:**
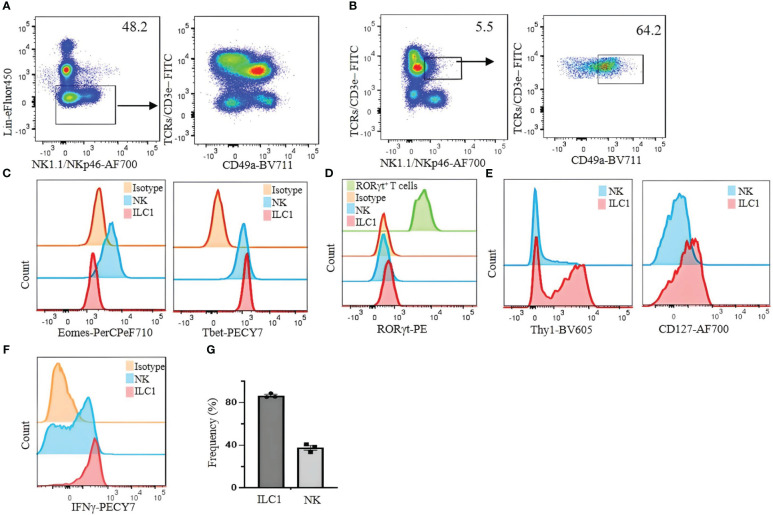
Limitations of CD49a as a selective marker for identifying liver ILC1s in C57BL/6 mice. **(A)** Lin^−^ cells were analyzed for the expression of CD49a on T cells. **(B)** NKT cells (TCRs/CD3ϵ^+^/NK1.1/NKp46^+^) were analyzed for CD49a expression. **(C)** Eomes and T-bet were analyzed on liver NK cells and ILC1s. **(D)** RORγt expression on liver NK cells and ILC1s; Lin^−^TCR/CD3ϵ^+^RORγt^+^ cells were used as positive control (green histogram). **(E)** Thy1 and CD127 expression on liver ILC1s and NK cells were analyzed. **(F)** Liver lymphocytes were incubated with phorbol 12-myristate 13-acetate (PMA)/ionomycin plus Brefeldin A for 4 hours and stained for intracellular IFNγ. CD49a^−^CD49b^+^ NK cells and CD49a^+^CD49b^−^ ILC1s were gated and analyzed for intracellular expression of IFNγ. Numbers indicate percentages of cells in indicated gates. **(G)** Frequency of IFNγ expressing ILC1 and NK cells. Data are shown as mean ± SEM.

**Figure 3:**
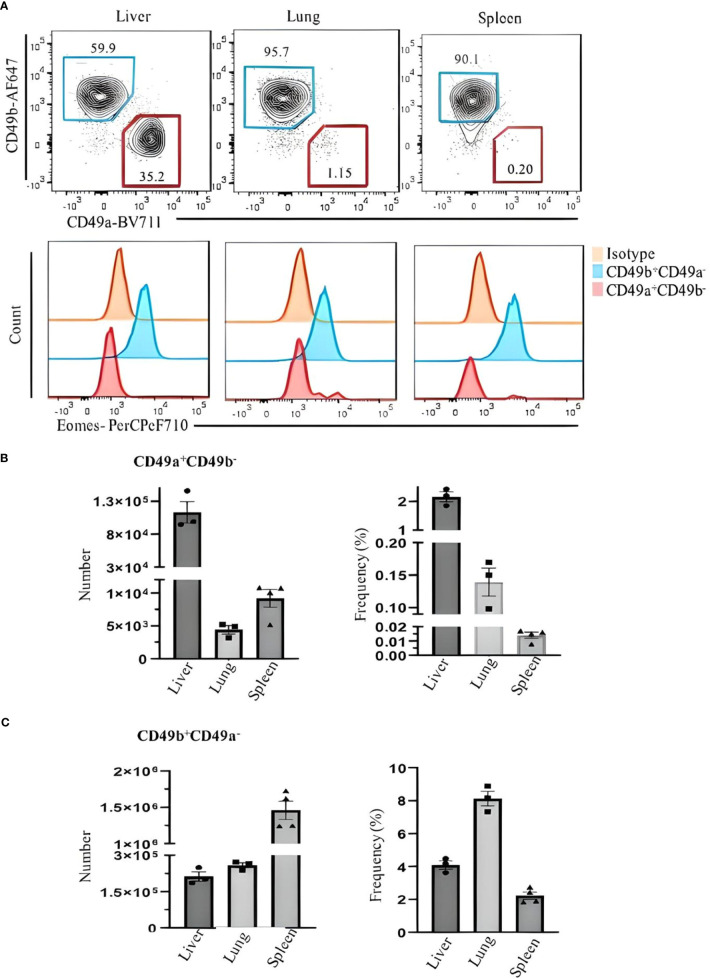
Comparing Eomes expression in ILC1s and NK cells in the liver, lung, and spleen of C57BL/6 mice. **(A)** Live CD45^+^Lin^−^NK1.1^+^NKp46^+^CD3ϵ/TCRs^−^ were gated and analyzed for CD49a and CD49b expression. Blue and red gates show CD49b^+^CD49a^−^ NK cells and CD49a^+^CD49b^−^ ILC1s, respectively. Numbers indicate percentages of cells in each gate. **(B)** Frequency and number of CD49a^+^CD49b^−^ and **(C)** CD49b^+^CD49a^−^ cells of CD45^+^ leukocytes in liver, lung, and spleen. Data are shown as mean ± SEM.

For our analysis of ILC1 in B6 mice, we used CD49b as an NK cell marker and CD49a as an ILC1 marker. However, we found that CD49a is not a specific marker of liver ILC1s, as other liver cells including some T cells and NKT cells also express CD49a (**Figures 2A, B**). Therefore, using a combination of markers is critical to define ILC1s. Lin^−^NK1.1^+^NKp46^+^CD3ϵ/TCRs^−^ cells can be divided into CD49b^+^CD49a^−^ NK cells and CD49b^−^CD49a^+^ILC1s. The former express Eomes and T-bet, whereas the latter express Eomes^−^T-bet^+^ (**Figure 2C**). Furthermore, both populations are RORγt^−^, indicating the absence of NKp46^+^ILC3 contamination (**Figure 2D**). Thy1 and CD127 are not reliable markers to distinguish ILC1s from NK cells, as some NK cells express Thy1 and CD127, while not all ILC1s are Thy1 and CD127 positive (**Figure 2E**). Approximately 80% (mean ± SEM) of ILC1s are positive for intracellular IFNγ (**Figure 2F**). This strategy is designed for B6 mouse liver ILC1s. However, defining ILC1s in other tissues remains a challenge. We found a small population of CD49a^+^ cells expressing Eomes in the lung and spleen (**Figure 3**). Therefore, it is critical to analyze the expression of transcription factors including Eomes and RORγt in addition to surface marker staining to define the ILC1 population in various tissues.

### 3.2 Analysis of ILC2s

**Figure 4:**
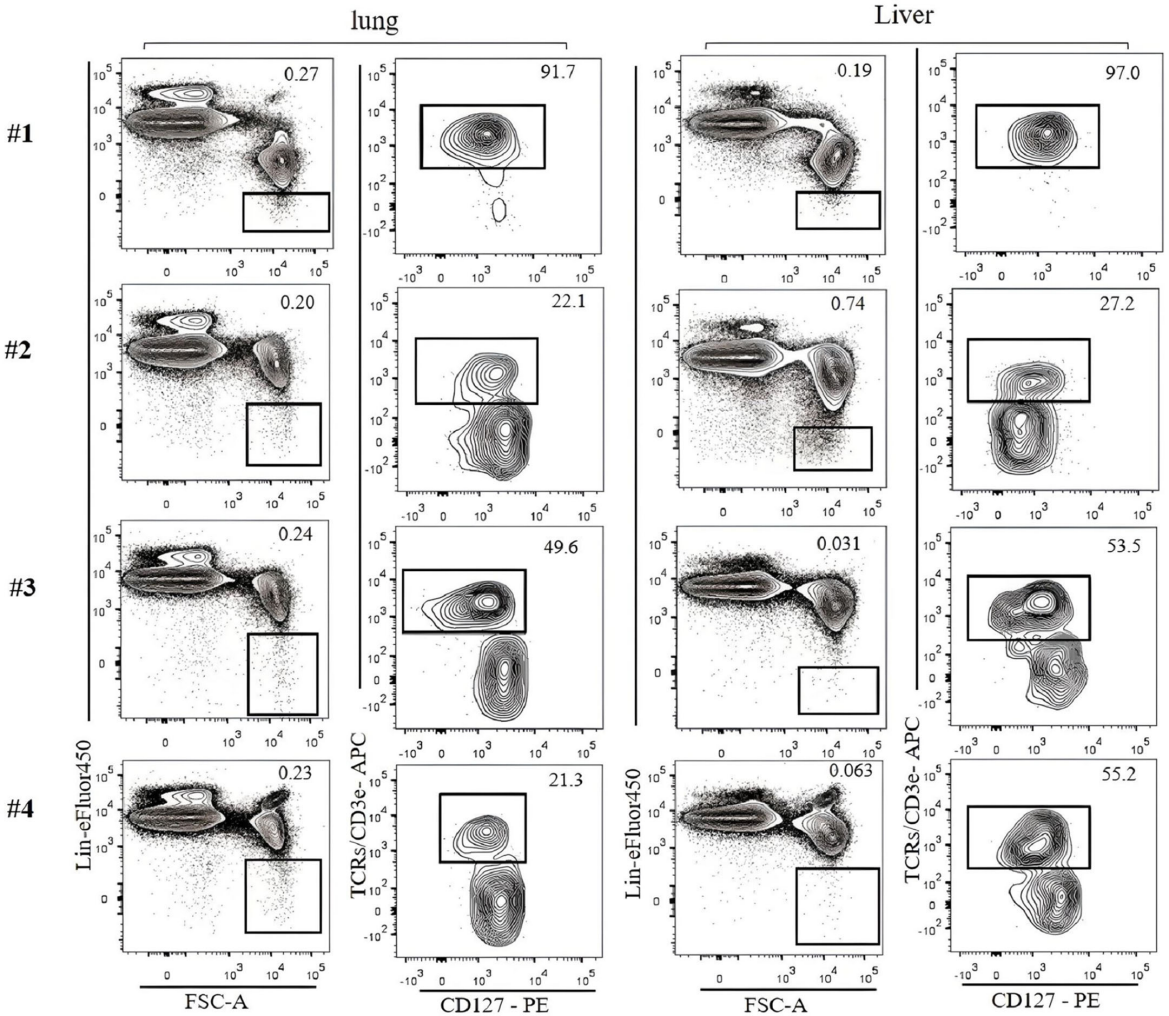
Lin-negative populations defined by different lineage cocktails were analyzed for T-cell contamination. ILC2s in the lung and the liver of C57BL/6 mice were analyzed. Gating was applied on live, single-cell, CD45^+^ leukocytes. The numbers on the left indicate Lin cocktails listed in **Table 3**. Numbers indicate percentages of cells in each gate.

Currently, ILC2s are defined by combinations of markers that vary among different studies. For example, lung ILC2s are defined by Lin^−^ST2^+^Thy1^+^CD25^+^ (17), Lin^−^CD45^+^, CD127^+^, ST2^+^ (18), CD45^+^CD3ϵ^−^CD19^–^CD127^+^CD25^+^ST2^+^ (19), and Lin^−^GATA3^+^ (20). Liver ILC2s are also defined by Lin^−^Sca-1^+^ST2^+^ (21), Lin^−^CD127^+^ST2^+^ (22), and Lin^−^CD45^+^GATA3^+^Thy1^+^ST2^+^ (23). In the literature, lung ILC2 numbers in naïve mice vary from approximately 2,000–3,000 cells (24, 25) to 8,000–15,000 (26) likely due to different strategies to identify ILC2s. One problem is the lack of consensus on which markers to include in the lineage cocktail among studies (**Table 3**). This can lead to the contamination of T cells, which have a similar phenotype as ILC2s (**Figure 4**), resulting in differences in the identification and enumeration of ILC2s. To avoid T-cell contamination, we propose to use T-cell markers in a separate channel from the lineage cocktail. This approach ensures that T cells are excluded from the ILC2 population.

**Table 3:** Lineage cocktails used in different studies.

#	Paper Title	Lineage cocktail	Ref
1	IL-17-producing ST2^+^ group 2 innate lymphoid cells play a pathogenic role in lung inflammation	CD3ϵ, B220, CD11b, CD11c, Gr-1, NK1.1, and FcϵRI	(27)
2	IL-33 promotes the egress of group 2 innate lymphoid cells from the bone marrow	CD3, CD5, CD45R (B220), CD11b, Gr-1 (Ly-6G/C), 7-4, and Ter-119	(28)
3	IL-25-responsive, lineage-negative KLRG1^hi^ cells are multipotential “inflammatory” type 2 innate lymphoid cells	CD3ϵ, CD5, CD19, B220, TCRγδ, NK1.1, CD11b, CD11c, Gr-1, FcϵR1, and TER119	(29)
4	A tissue checkpoint regulates type 2 immunity	B220, CD11b, CD19, TCRgd, CD11c, CD3, CD5, Ter 119, NK 1.1, and CD8	(30)

**Figure 5:**
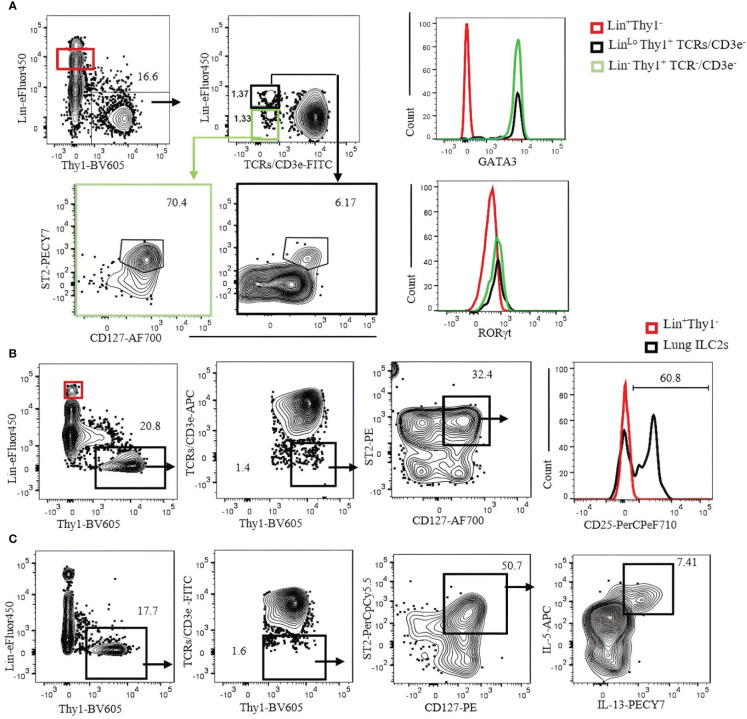
Characterization of C57BL/6 mouse lung ILC2s with CD3ϵ/TCR separated from the Lin cocktail. **(A)** Lin-negative/low Thy1^+^ cells were gated, and TCR/CD3ϵ^−^ cells were further divided into Lin^−^ (green gate) and Lin^lo^ (bold black gate). ILC2s in each gate were identified by CD127^+^ST2^+^ and confirmed by GATA3^+^RORγt^−^ (right histograms). Lin^+^ population (red gate) was used as negative control. **(B)** Lung ILC2s were gated by Lin^−^/low Thy1^+^TCR/CD3ϵ^−^ST2^+^CD127^+^ and analyzed for CD25 expression. Lin^+^ population (red gate and red histogram) was used as negative control. **(C)** Lung lymphocytes were incubated with phorbol 12-myristate 13-acetate (PMA)/ionomycin and Brefeldin A for 3 hours. ILC2s (Thy1^+^Lin^−^TCR/CD3ϵ^−^ST2^+^CD127^+^) were analyzed for intracellular IL-5 and IL-13. Gating was applied on live, single-cell, CD45^+^ leukocytes. Numbers indicate percentages of cells in each gate.

To identify ILC2s in the lung, we used a Lin cocktail of CD19, Ter119, B220, NK1.1, NKp46, GR-1, CD11b, and CD11c labeled with eFluor450 combined with CD3ϵ/TCRs labeled with fluorescein isothiocyanate (FITC) or allophycocyanin (APC). ILC2s are then gated by Lin^−^Thy1^+^CD3ϵ/TCRs^−^CD127^+^ST2^+^. The number of ILC2s in the naïve B6 mouse lungs identified by this strategy is approximately 3,000 cells per mouse. It should be noted that not all ILC2s in the lung are Lin negative, but some of them seem to weakly express Lin markers (**Figure 5A**, contour plots). ILC2s thus identified can be confirmed by transcription factor staining (GATA3^+^RORγt^−^) (**Figure 5A**, histograms). Most (approximately 80%) lung ILC2s (Lin^−^Thy1^+^CD3ϵ/TCRs^−^ST2^+^GATA3^+^) also express CD25 (**Figure 5B**). Intercellular cytokines in ILC2s are analyzed by the expression of IL-5 and IL-13 in the same gate following a short-term (3 hours) incubation with PMA/ionomycin in the presence of Brefeldin A. Approximately 7% (mean ± SEM) of ILC2s express intracellular IL-5 and IL-13 (**Figure 5C**).

**Figure 6:**
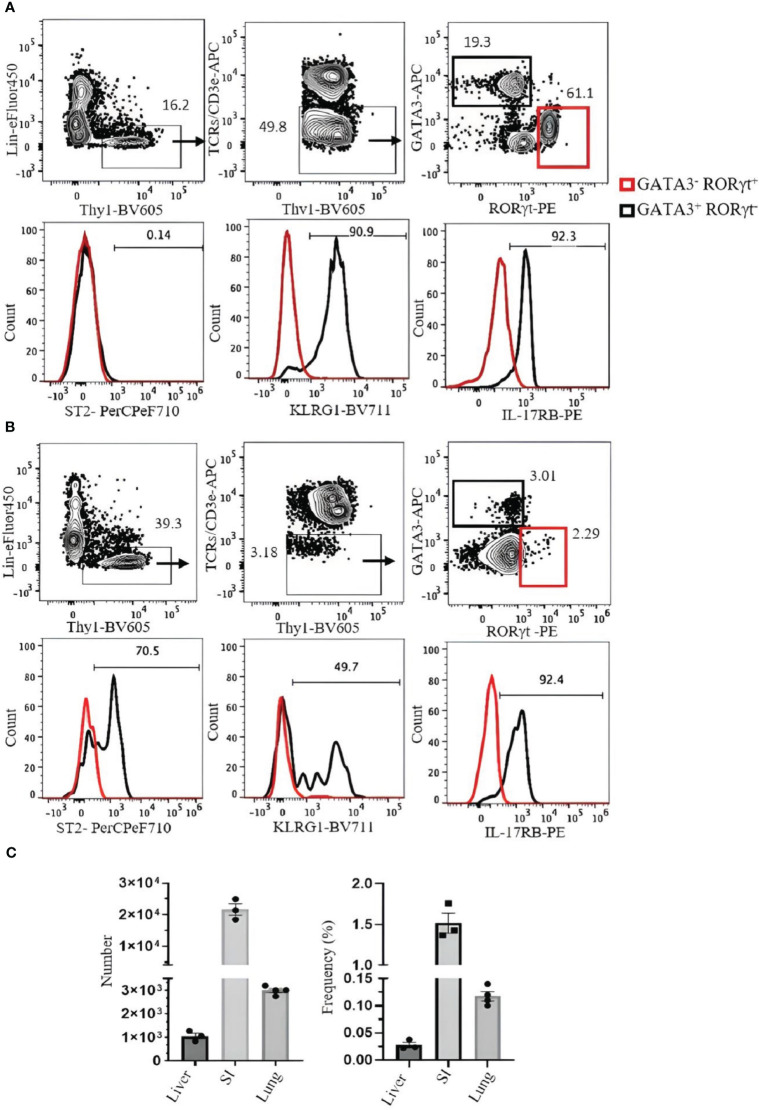
Analysis of ILC2s in the small intestine (SI) and liver of C57BL/6 mouse. **(A)** ILC2s in the SI were gated as Thy1^+^Lin^−^TCRs/CD3ϵ^−^ST2^+^GATA3^+^RORγt^−^, and RORγt^+^ILC3s (red square) were used as negative control. Histograms show the expression of ST2, KLRG1, and IL-17RB on ILC2s. **(B)** ILC2s in the liver were gated as Thy1^+^Lin^−^TCRs/CD3ϵ^−^GATA3^+^RORγt^−^. RORγt^+^ILC3s (red square) were used as negative control. Histograms show the expression of ST2, KLRG1, and IL-17RB. Gating was applied on live, single-cell, CD45^+^ leukocytes. Numbers indicate percentages of cells in each gate. **(C)** Frequency and number of ILC2s (of CD45^+^ leukocytes). Data are shown as mean ± SEM.

SI ILC2s (Lin^−^hy1^+^CD3ϵ/TCRs^−^GATA3^+^RORγt^−^) are ST2^−^, but approximately 90% express KLRG1, and over 90% are IL-17RB positive (**Figure 6A**). Liver ILC2s (Lin^−^Thy1^+^CD3ϵ/TCRs^−^GATA3^+^RORγt^−^) can be divided into ST2^+^ (approximately 70%) and ST2^−^ populations. Approximately 50% of them are KLRG1^+^, and over 90% are IL-17RB^+^ (**Figure 6B**). The frequency and number of ILC2s in the liver, SI, and lung are shown in **Figure 6C**.

### 3.3 Analysis of ILC3s

**Figure 7:**
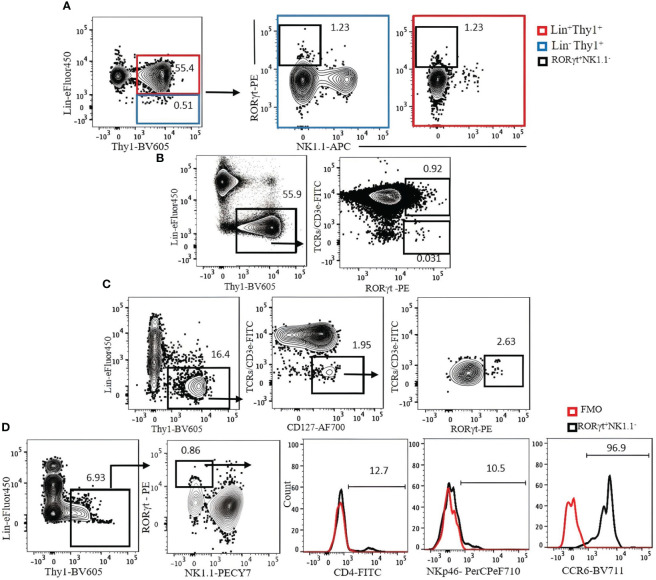
**(A)** The expression of Lin markers on RORγt^+^NK1.1^−^ cells (bold black square) in C57BL/6 mouse lung. Lin cocktail included TCR/CD3ϵ, CD11b, CD11c, GR-1, B220, and Ter119. Lin^+^ cells (red square) were used as positive control. Gating was applied on live, single-cell, CD45^+^ leukocytes. **(B)** The expression of RORγt on T cells. **(C)** ILC3s in C57BL/6 mouse lung were identified by serial gating as shown. **(D)** Expression of CD4, NKp46, and CCR6 on Rag1^−/−^ mouse lung ILC3s. Fluorescence minus one (FMO) was used as control negative. Gating applied on live, single, CD45^+^Lin^−^Thy1^+^NK1.1^−^ cells. Lin cocktail included Ter119, B220, CD11c, CD11b, and Gr-1. Numbers indicate percentages of cells in each gate.

**Figure 8:**
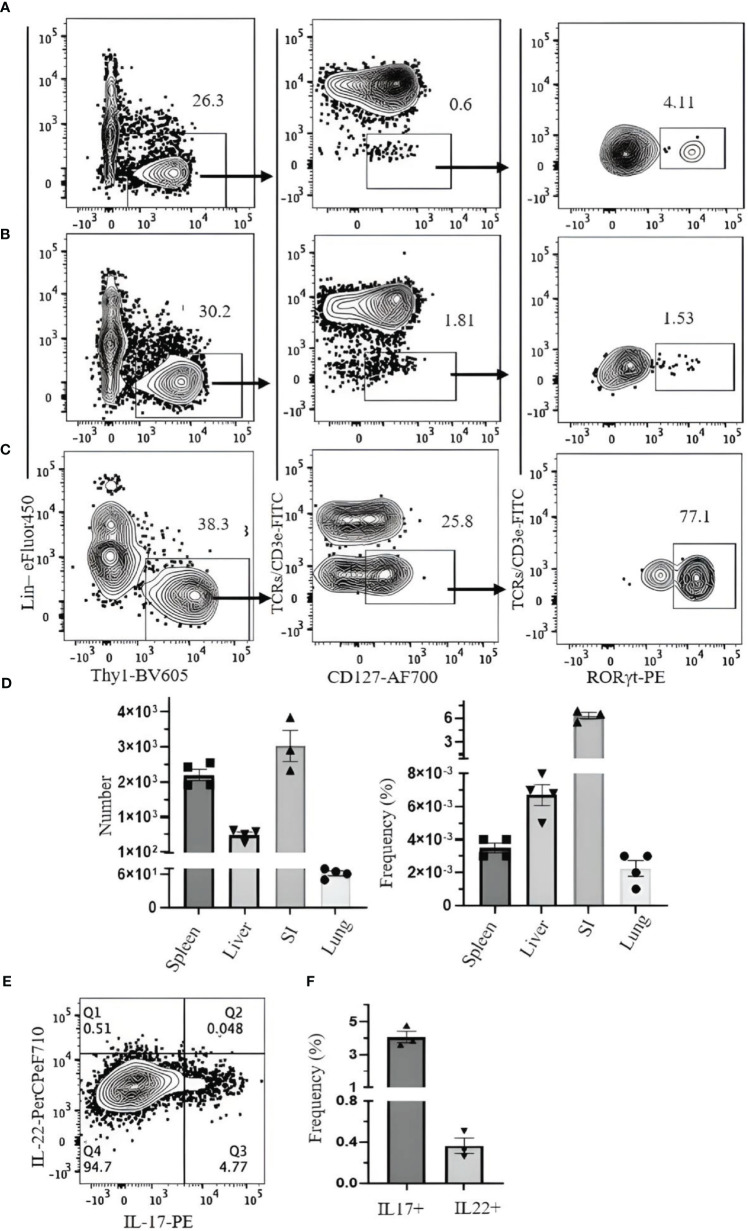
Gating strategy for ILC3 characterization in C57BL/6 mouse. Lymphocytes were isolated from **(A)** spleen, **(B)** liver, and **(C)** small intestine. **(D)** Frequency and number of ILC3s (of CD45^+^ leukocytes) in spleen, liver, small intestine (SI), and lung. **(E)** SI lymphocytes were incubated with phorbol 12-myristate 13-acetate (PMA)/ionomycin and Brefeldin A for 3 hours. ILC3s (Thy1^+^Lin^−^TCR/CD3ϵ^−^CD127^+^Rorgt^+^) were analyzed for intracellular IL-17 and IL-22. **(F)** The frequency of IL-17- and IL-22-producing ILC3s. Data plotted are means ± SEM. Gating applied on live CD45^+^lin^−^Thy1^+^TCRs/CD3ϵ^−^CD127^+^RORγt^+^ cells. Lin cocktail included Ter119, CD19, B220, CD11c, CD11b, GR-1, and NK1.1. Numbers indicate percentages of cells in each gate.

We investigated strategies to identify ILC3s in the lung, spleen, liver, and SI. ILC3s are similar to other ILCs in terms of surface markers and are Lin^−^Thy1^+^CD127^+^. Because some ILC3s express NKp46, the Lin cocktail for ILC3s excludes this marker. There is no ILC3-specific surface marker, and it is difficult to discriminate them from other ILCs by cell surface markers alone. Therefore, staining for RORγt, which is expressed in ILC3s but not in other ILCs, is required to specifically identify ILC3s. When CD3ϵ/TCR are included in the Lin cocktail for analysis of ILC3s in the naïve B6 mouse lungs, there is no clear separation between Lin^+^ and Lin^−^ populations, and both populations include RORγt^+^ cells (**Figure 7A**). When TCR/CD3ϵ is in separate fluorochrome, it became clear that some T cells express RORγt (**Figure 7B**). ILC3s in naïve C57BL/6 lung are a very rare population, but they could be identified by Lin^−^Thy1^+^CD127^+^CD3ϵ/TCR^−^RORγt^+^ (**Figure 7C**). Rag1-ko mice have more ILC3s in the lung than wild-type B6 and can be divided into CD4^+^, NKp46^+^, and CCR6^+^ subsets (**Figure 7D**). ILC3s in the spleen, liver, and SI were also detected by Lin^−^Thy1^+^CD127^+^CD3ϵ/TCR^−^RORγt^+^ (**Figures 8A-D**). Approximately 4% and 0.4% (mean ± SEM) of ILC3s are IL-17 and IL-22 positive, respectively (**Figures 8E, F**).

**Figure 9:**
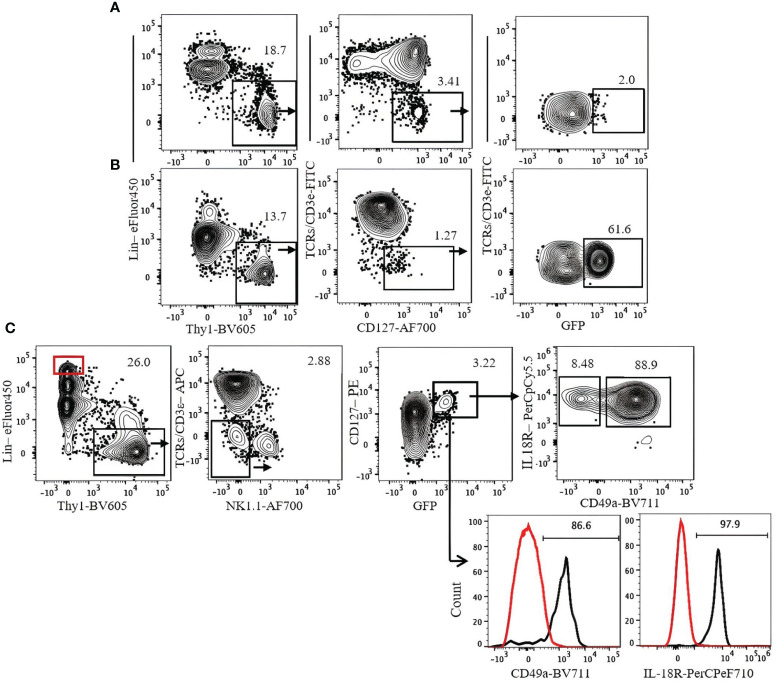
Gating strategy for ILC3 characterization in *Rorc(γt)-*eGFP mice. **(A)** Lung. **(B)** Spleen. **(C)** Expression of CD49a and IL-18R on lung ILC3s. Lin^+^Thy1^−^ cells (red gate and red histogram) were used as control. Gating applied on live CD45^+^lin^−^Thy1^+^TCRs/CD3ϵ^−^NK1.1^−^CD127^+^GFP^+^ cells. Lin cocktail included Ter119, CD19, B220, CD11c, CD11b, and GR-1. Numbers indicate percentages of cells in each gate.

Transcription factor staining requires fixation and permeabilization steps. To analyze live ILC3s, we used heterozygous *Rorc(*γ*t)*-eGFP reporter mice. ILC3s in these mice can be detected by Lin^−^Thy1^+^NK1.1^−^CD3ϵ/TCR^−^CD127^+^GFP^+^. ILC3s are mostly IL-18R^+^ and CD49a^+^ (**Figure 9**).

## 4 Discussion

The ILC population is heterogeneous, and currently, there is no single way to identify all ILCs in various tissues. This study has focused on flow cytometric analyses of ILC1s in the liver, ILC2s in the lung and liver, and ILC3s in the lung, spleen, and SI. The common first step to identify ILCs is to exclude myeloid cells, erythroid cells, dendritic cells, T cells, and B cells based on Lin marker expression. The Lin cocktail commonly includes T-cell markers (CD3ε/TCRs), and the Lin^−^ population is expected to be T cell-free. However, in our analyses, T-cell contamination is still the biggest potential problem in identifying ILCs (7, 31). Lin^−^ cells are not clearly separated from Lin^+^ cells, and some ILCs seem to weakly express some Lin markers. Therefore, Lin^−^ gate is ambiguous and subjective, and it is difficult to exclude all T cells from the Lin^−^ population. We have found that the best way to avoid T-cell contamination is not to include CD3ε/TCRs in the Lin cocktail but to have them in a different fluorochrome instead. In this way, T cells can be more clearly excluded. Because apparent ILC functions can be attributed to contaminating T cells, it is critical to specifically exclude all T cells. In conventional flow cytometry, having CD3ε/TCRs in separate fluorochrome from the Lin cocktail reduces the capability of other marker analyses due to the limited number of fluorescence combinations. This can be resolved by the use of a spectral flow cytometer (32).

For ILC1 analysis, distinguishing them from NK cells and other ILCs especially ILC3s is necessary. In our current study, liver ILC1s are identified by Lin^−^NKp46^+^NK1.1^+^CD49a^+^CD49b^−^. The combination of CD49a and CD49b clearly separates ILC1s from NK cells, whereas NK1.1 expression excludes ILC3s. This can be confirmed by the expression of the NK-associated transcription factor Eomes in CD49a^−^CD49b^+^, while the CD49a^+^CD49b^−^ population lacks Eomes and the ILC3-associated transcription factor RORγt. It should be noted that CD49a is expressed not only on ILC1s but also on other lymphocytes, including ILC3s and NKT cells, and it is important to have CD3ϵ/TCR in separate fluorochrome to avoid contamination of T cells as discussed above. This strategy cannot be universally applied to ILC1s in all tissues and other strains of mice lacking NK1.1. It has been reported that salivary gland ILC1s express CD49b and Eomes, and intestinal ILC1s also express Eomes (9).

ILC2s in the lung are identified by Lin^−^CD127^+^Thy1^+^ST2^+^. We have shown that unless those cells are separately stained for CD3ϵ/TCRs, it is very difficult to avoid T-cell contamination. While ILC2s in the lung are efficiently identified by this strategy, a minor population of lung ILC2s termed inflammatory ILC2s is known to be ST2^−^ (33). CD25 can be used to identify lung ILC2s, but not all lung ILC2s express CD25. The majority of ILC2s in the SI do not express ST2 (24), and KLRG1 may be used to identify them instead. However, approximately 10% of SI ILC2s are KLRG1 negative.

Approximately 30% of Liver ILC2s do not express ST2. For those, IL-17RB (IL-25R) can be used in place of ST2 to identify ILC2s. However, recently reported IL-18R^+^ ILC progenitors also express IL-17RB (34), and IL-18R^+^ cells have to be excluded from the analysis of ST2^−^ ILC2s.

Activated lung ILC2s may downregulate CD127 and ST2. Cavagnero K et al. reported ST2^+^CD127^−^ and ST2^−^CD127^−^ cells in the lung of *Alternaria*-treated mice (35). Staining for GATA3, RORγt, T-bet, and Eomes should determine their identity.

ILC3s share many surface markers with other ILCs; to identify and distinguish ILC3s from other ILCs, analysis for RORγt expression is critical. RORγt expression also distinguishes ILC3s from the IL-18R^+^ ILC progenitors discussed above. While both are IL-18R^+^, ILC3s but not ILC progenitors are RORγt^+^. RORγt expression can be analyzed by nuclear staining of fixed and permeabilized cells or by the *Rorc(*γ*t)*-eGFP reporter mice (13). The latter allows the identification of live ILC3s. Because homozygous *Rorc(*γ*t)*-eGFP mice are RORγt-deficient, only heterozygous mice can be analyzed for ILC3s. Due to a half-dose of RORγt in the heterozygous mice, ILC3 numbers in the heterozygous mice are lower than wild type. Because RORγt is also expressed in some T cells, T cells have to be excluded from ILC3 analyses by the strategy described above for ILC1s and ILC2s. A subpopulation of ILC3s also expresses NKp46 but not NK1.1, while NK cells and ILC1s express both. In our study, NK1.1 was used to discriminate ILC3s from ILC1s and NK cells.

In summary, the strategies to identify ILCs have to be designed for each ILC population and tissue due to the heterogeneity of ILCs. In all cases, it is critical to exclude T cells, and the identity of ILCs should be confirmed by a combination of transcription factors GATA3, RORγt, T-bet, and Eomes.

## Data availability statement

The original contributions presented in the study are included in the article/supplementary material. Further inquiries can be directed to the corresponding author.

## Ethics statement

The use of these mice was approved by the animal committee of the University of British Columbia and in accordance with the guidelines of the Canadian Council on Animal Care. The study was conducted in accordance with the local legislation and institutional requirements.

## Author contributions

MS, DK, and MO wrote and edited the manuscript. MS, DK, and MO performed the experiments and generated figures and tables. FT reviewed the drafts, provided critical input, and edited the manuscript and figures. All authors contributed to the article and approved the submitted version.
